# Transcriptome Adaptation of Group B *Streptococcus* to Growth in Human Amniotic Fluid

**DOI:** 10.1371/journal.pone.0006114

**Published:** 2009-07-01

**Authors:** Izabela Sitkiewicz, Nicole M. Green, Nina Guo, Ann Marie Bongiovanni, Steven S. Witkin, James M. Musser

**Affiliations:** 1 Center for Molecular and Translational Human Infectious Diseases Research, The Methodist Hospital Research Institute, and Department of Pathology, The Methodist Hospital, Houston, Texas, United States of America; 2 Weill Medical College of Cornell University, New York, New York, United States of America; Max Planck Institute for Infection Biology, Germany

## Abstract

**Background:**

*Streptococcus agalactiae* (group B *Streptococcus*) is a bacterial pathogen that causes severe intrauterine infections leading to fetal morbidity and mortality. The pathogenesis of GBS infection in this environment is poorly understood, in part because we lack a detailed understanding of the adaptation of this pathogen to growth in amniotic fluid. To address this knowledge deficit, we characterized the transcriptome of GBS grown in human amniotic fluid (AF) and compared it with the transcriptome in rich laboratory medium.

**Methods:**

GBS was grown in Todd Hewitt-yeast extract medium and human AF. Bacteria were collected at mid-logarithmic, late-logarithmic and stationary growth phase. We performed global expression microarray analysis using a custom-made Affymetrix GeneChip. The normalized hybridization values derived from three biological replicates at each growth point were obtained. AF/THY transcript ratios representing greater than a 2-fold change and P-value exceeding 0.05 were considered to be statistically significant.

**Principal Findings:**

We have discovered that GBS significantly remodels its transcriptome in response to exposure to human amniotic fluid. GBS grew rapidly in human AF and did not exhibit a global stress response. The majority of changes in GBS transcripts in AF compared to THY medium were related to genes mediating metabolism of amino acids, carbohydrates, and nucleotides. The majority of the observed changes in transcripts affects genes involved in basic bacterial metabolism and is connected to AF composition and nutritional requirements of the bacterium. Importantly, the response to growth in human AF included significant changes in transcripts of multiple virulence genes such as adhesins, capsule, and hemolysin and IL-8 proteinase what might have consequences for the outcome of host-pathogen interactions.

**Conclusions/Significance:**

Our work provides extensive new information about how the transcriptome of GBS responds to growth in AF, and thus new leads for pathogenesis research.

## Introduction

Intrauterine and postpartum infections remain an important cause of morbidity and mortality worldwide. One of the bacterial species commonly responsible for these infections is *Streptococcus agalactiae*, also known as group B *Streptococcus* (GBS). GBS colonizes the urogenital or gastrointestinal tract of about 10%–30% of humans, depending on gender, geographical origin, ethnicity, and screening method used (for a review see [1] and references therein). In recent decades GBS has become an important human pathogen [2], now responsible for a large percentage of female urogenital tract infections in non-pregnant women and amnionitis and septic abortion in pregnant individuals [3], [4]. GBS also is a major cause of fatal septicemia and meningitis in newborns and invasive infections in elderly and people with underlying diseases [3], [4]. Recent studies [5], [6], [7] have shown that the transcriptome of GBS responds extensively to environmental changes, therefore we hypothesized that this organism will significantly remodel its global transcript profile in response to growth in human amniotic fluid. In this study, we employed an *ex vivo* strategy to characterize the global transcriptome response of GBS when grown in human amniotic fluid. The *ex vivo* strategy has been successfully used to study adaptation of pathogenic bacteria to multiple environments of the human body such as blood and saliva [7], [8], [9]. To gain significant new information about the interaction of GBS with amniotic fluid over time, we conducted expression microarray analysis at three time points throughout the pathogen growth cycle.

## Materials and Methods

### Bacterial strains and routine growth

Serotype III GBS strain NEM316 was used in these studies because the genome has been sequenced, the organism has been used in many pathogenesis studies, and serotype III organisms cause a large number of serious human infections [10]. The strain was grown in Todd Hewitt medium with 0.5% yeast extract (THY) or on Trypticase Soy agar (TSA) II plates supplemented with 5% sheep blood (BD Diagnostics) at 37° in a 5% CO_2_ atmosphere.

### Growth of GBS in human amniotic fluid

Human amniotic fluid (AF) was collected from pregnant women seen at The Methodist Hospital, Houston, Texas, or Weill Medical College of Cornell University in New York City. Samples were collected in accordance with an exempt human subjects protocol approved by the Institution Review Board of each institution. The study involved collection of existing diagnostic specimens routinely collected during clinical procedures as amniocenteses and would have been otherwise discarded. Specimens were stripped of all identifiers and processed in a manner that subjects cannot be directly or indirectly identified.

**Figure 1:**
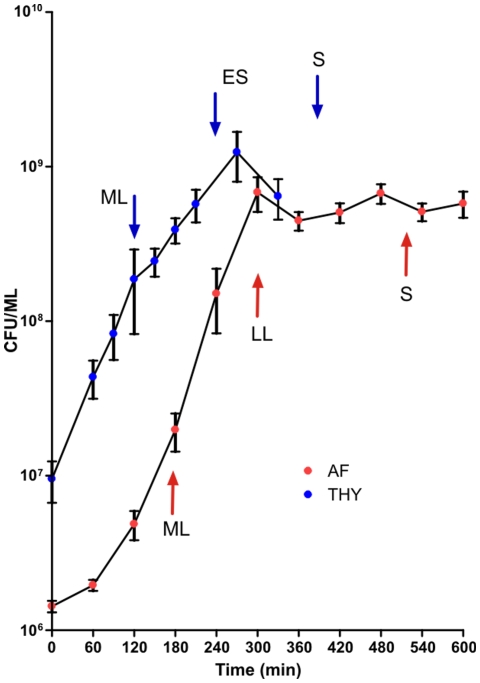
Growth of GBS in THY and AF. Growth of GBS in THY laboratory medium (blue) and human AF (red). Arrows mark time points of sample collection for RNA isolation. Blue arrows denote points for samples collected during growth in THY, and red arrows denote collection points for bacteria grown in AF. ML, mid-logarithmic growth phase, LL; late-logarithmic growth phase, ES; early stationary growth phase; and S, stationary growth phase.

After collection, each specimen was centrifuged to remove host cells, filter sterilized, and frozen at −20°C. After thawing, each AF sample was tested to determine if it supported growth of GBS. Aliquots (250 µl) of heat inactivated (95° for 5 min) AF were inoculated with GBS prepared as follows. Bacteria from overnight cultures grown in THY were collected by centrifugation, washed twice with sterile PBS, and suspended in PBS. 10 µl of 100× bacteria diluted further in PBS were used to inoculate each 250 µl sample of AF (resulting in a final inoculum of ∼10^4^ CFU/ml) and were incubated at 37°C, in 5% CO_2_ for 24 h. To avoid artifacts caused by carryover of THY medium, after 24 h of growth in AF (designated AF1), the GBS were diluted 1∶500 into a fresh aliquot of AF (designated AF2). Growth of GBS in AF2 was quantified every hour for first 12 h and thereafter every 12 h by plating serial dilutions on TSA II plates (BD Diagnostics). For transcriptome studies, AF samples were pooled and three independent AF2 cultures were inoculated with GBS (biological replicates). Bacteria were collected by centrifugation at time points corresponding to the mid-logarithmic (ML), late-logarithmic/early stationary (LL) and stationary (S) phase of growth (Fig. 1).

Cultures grown in THY medium were prepared as described previously [5]. Briefly, three independent cultures of GBS were grown in the same lot of THY broth and GBS cells were harvested at three time points corresponding to mid-logarithmic (ML), late log/early stationary (ES), and stationary (S) growth phase (Fig. 1).

### RNA isolation and processing

The bacterial aliquots used for RNA isolation were mixed with 2 volumes of RNA Protect reagent (Qiagen), and the cells were collected by centrifugation and stored at −80°C. RNA was isolated using a modified TRIZOL (Invitrogen) method [11]. Briefly, GBS pellets were suspended in 200 µl of Max Bacterial Enhancement Reagent (Invitrogen), incubated according to the manufacturer's recommendations, mixed with 1 ml of TRIZOL, and disrupted using lysis matrix B (MP Biochemicals). Cell debris was removed by centrifugation, and RNA was extracted with chloroform and precipitated with isopropanol. The precipitated RNA was suspended in 100 µl of RNAse free water (Ambion, Austin TX) and further purified using RNeasy 96 well plates. All samples were processed simultaneously to minimize experimental variation. Reverse transcription, cDNA fragmentation, and labeling was performed as described previously [12].

### Microarray analysis

Microarray analysis was performed using a custom-made Affymetrix chip that contained 1,994 probe sets, representing the annotated ORFs of GBS strain NEM316 [13]. Chip hybridization data were acquired using Affymetrix GeneChip Operating Software (GCOS 1.4) and normalized to allow multi-condition comparison. GCOS-acquired hybridization intensity values were normalized to the total intensity of all GBS genes present on the chip. Individual intensity for the gene transcripts generated by GCOS was divided by the sum of all intensities of the GBS hybridizing probes. Normalized hybridization values were used in all subsequent analysis. Data derived from three biological replicates obtained from three independent cultures were used to calculate mean values. PartekPro (Partek) and Array Assist (Stratagene) software were used to assess chip quality and chip-to-chip variability, and for data mining and visualization. Average normalized hybridization values were used to calculate AF/THY transcript ratios. Average values generated after hybridization of samples from AF_ML phase were divided by values generated from samples THY_ML to generate mid-logarithmic AF/THY ratios (ML). Average values from samples AF_LL were divided by values generated from samples THY_ES to generate late-logarithmic AF/THY ratios (LL). Average values from samples AF_S were divided by values generated from samples THY_S to generate stationary-phase AF/THY ratios (S). Only AF/THY ratios above a 2-fold change and with a P value less than 0.05 were included in the functional analysis. Normalized hybridization values are deposited in GEO database (http://www.ncbi.nlm.nih.gov/geo/) under GSE14456 and GSE12238 accession numbers.

## Results and Discussion

### Characterization of GBS growth in AF

Prior to characterizing the transcriptome of GBS grown in human AF, we studied the growth of strain NEM316 in THY medium. GBS grew rapidly in this medium, with a generation time of ∼35 min in logarithmic phase (Fig. 1). The bacterial density reached ∼10^9^ CFUs/ml in the stationary phase (Fig. 1). We next analyzed the growth of strain NEM316 in human AF. AF has been reported to have antimicrobial properties toward various species of bacteria due to β-lysin and lysozyme activity, which depends on divalent cations such as zinc and phosphate [14], [15]. In multiple studies reported in the literature 18–73% of AF samples exhibited inhibitory properties towards various bacterial species. The antimicrobial properties depend on gestation stage and ethnicity [16], [17], [18], [19], [20]. Because individual specimens of AF can vary in their antimicrobial properties even towards GBS [21], we first tested the ability of the collected samples to support GBS growth. Consistent with the majority of the data reported in the literature, none of the AF specimens significantly inhibit the growth of strain NEM316 (data not shown). To avoid the effects of sample-to-sample variability, pooled AF was used in all subsequent experiments. Growth of GBS in AF is comparable with growth in THY with respect to cell density (∼10^9^ CFU/ml) and growth rate in exponential phase (Fig. 1).

### Expression microarray analysis: quantitative differences during growth and in response to AF

**Figure 2:**
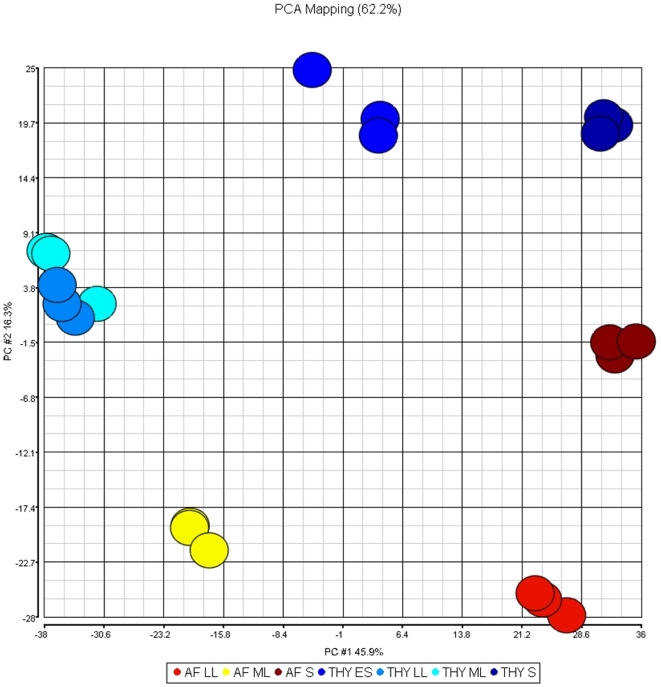
PCA plot analysis of microarray data. Each circle represents a single biological replicate. Each experimental condition is designated with a separate color. Distinct clusters of three replicates denote highly reproducible arrays. ML, mid-logarithmic growth phase, LL; late-logarithmic growth phase, ES; early stationary growth phase; and S, stationary growth phase.

**Figure 3:**
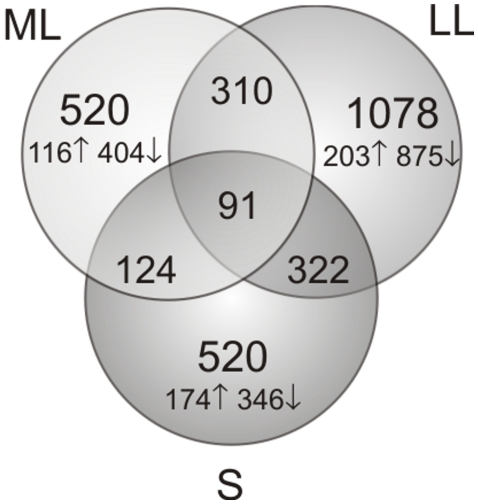
Quantitative differences in gene expression between THY and AF. Genes regarded as differentially expressed have an AF/THY transcript level ratio of 2 and above (better expressed in AF) or 0.5 and below (better expressed in THY). Arrow up, number of genes better expressed in AF; arrow down, number of genes better expressed in THY. ML, mid-logarithmic growth phase, LL; late-logarithmic growth phase, ES; early stationary growth phase; and S, stationary growth phase.

To characterize the transcriptome of GBS grown in AF, we used an *ex vivo* expression microarray analysis strategy that we previously employed to study transcription interactions of streptococci with body fluids such as blood and saliva [7], [8], [9]. Strain NEM316 was grown in THY broth or pooled AF and harvested at various time points (Fig. 1). After transcriptome data acquisition, we assessed chip-to-chip data variability and quality using principal component analysis (PCA) (Fig. 2). The PCA analysis discriminated very well between the transcript data from the chips representing the various growth phases studied (Fig. 2). These results indicated that the transcriptome profile data from triplicate experiments were highly reproducible and of sufficient quality to permit robust statistical analysis and interpretation. The data clearly show that the transcriptome of GBS strain NEM316 is considerably remodelled in a growth-phase and growth medium-specific fashion. During growth of GBS in either THY [5] or AF (this work), we observed over 70% of all transcripts (table S1) exhibiting differential expression during at least one experimental growth phase what is a sign of great transcriptome plasticity in response to environment changing over time. Interestingly the biggest differences between expression in AF and THY are observed during transition from logarithmic to stationary phase (LL) (Fig. 3) and as many as 54% of all GBS transcripts present on the array are differentially expressed.

**Figure 4:**
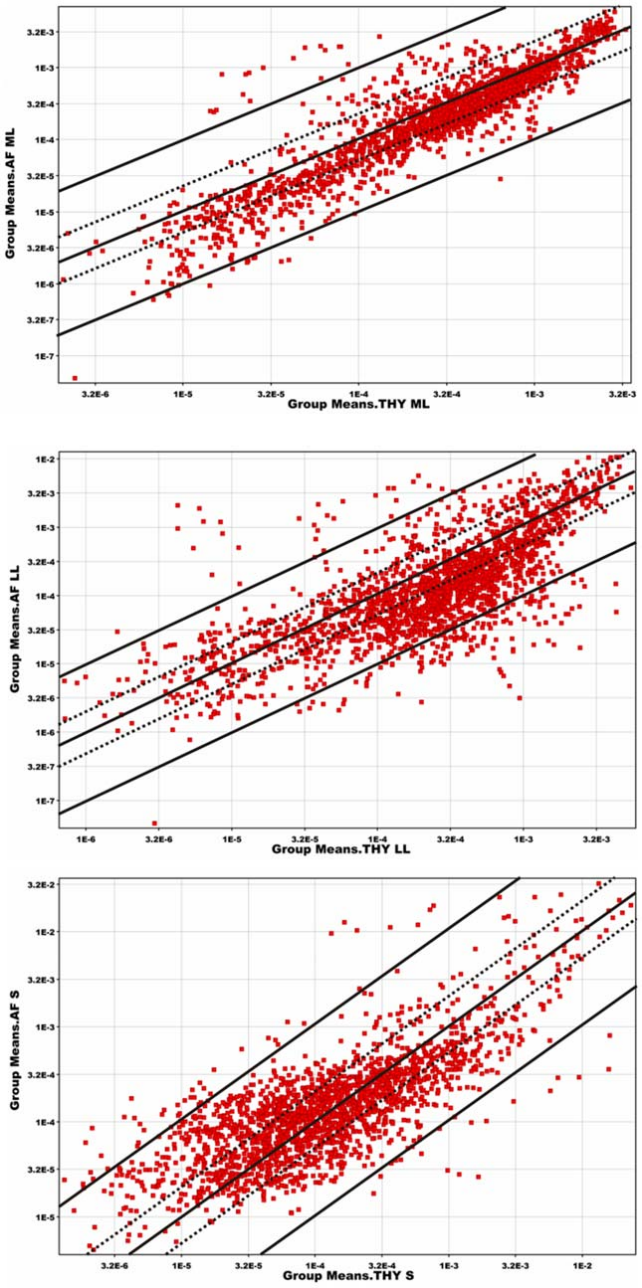
Dynamics of gene expression between THY and AF at various stages of growth. The location of each dot represents a single transcript. Dotted lines denote two-fold difference between transcript level in AF and THY, thick lines denote ten-fold difference in expression between AF and THY. Each panel represents differences in expression in mid-logarithmic (ML), late-logarithmic (LL) and stationary (S) growth phases. *x*-axis, expression level in THY; *y*-axis, expression level in AF.

We next used a scatter plot analysis (Fig. 4) to compare the dynamics of transcript expression in ML, LL, and S phase between strain NEM316 grown in AF and THY. In the ML phase, transcript changes were rather modest, comparing with changes in LL and S phases, with AF/THY ratios rarely exceeding 10-fold. In the ML and LL phase transcripts are shifted toward THY, with a smaller number of transcripts expressed better in AF. In the S phase of growth, the general level of transcription is lower (note shift of spots towards left bottom corner, Fig. 4) than in ML and LL phase. However, the number of genes up regulated in AF is higher than in other phases of growth.

**Figure 5:**
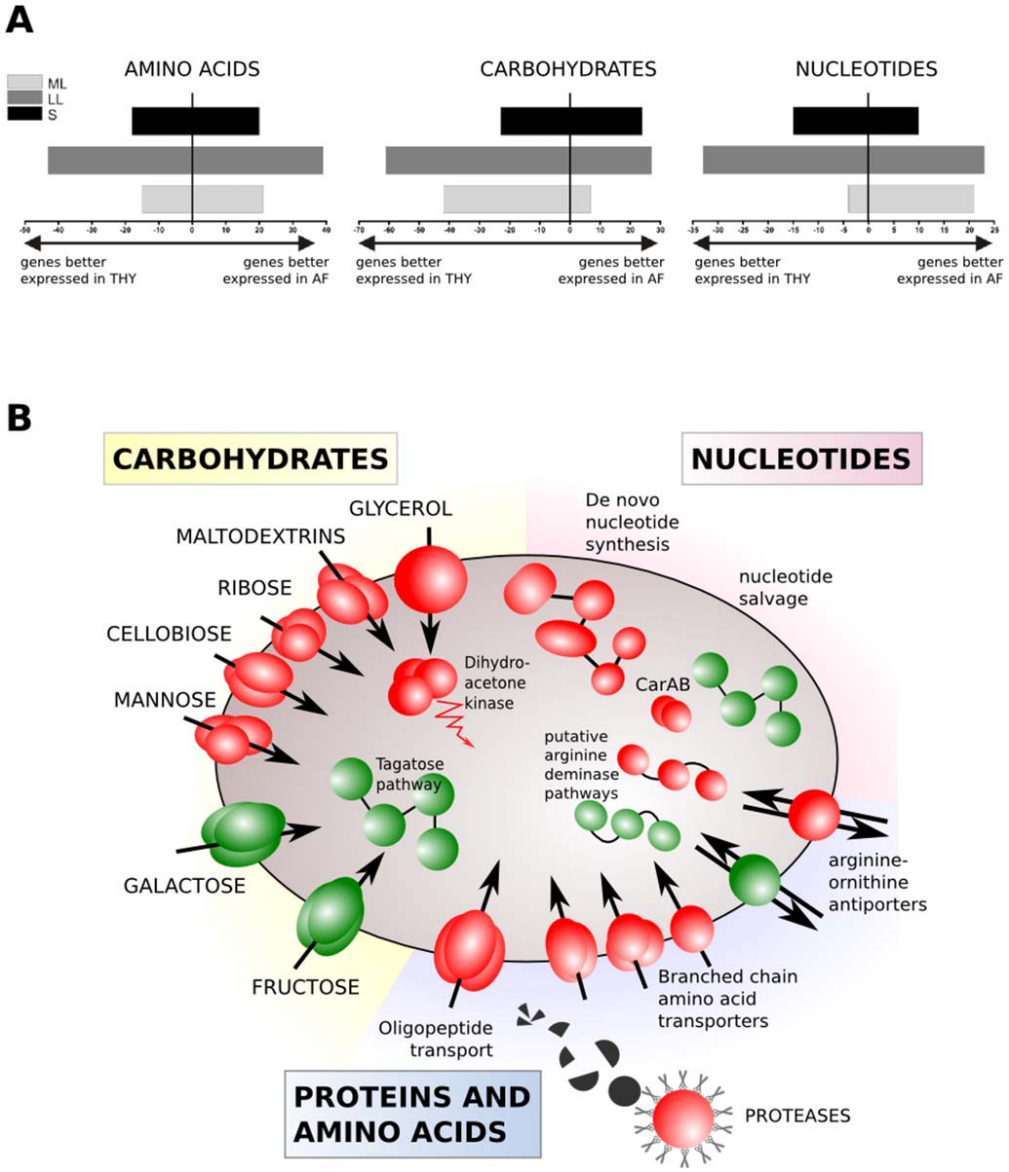
Functional dynamics of transcriptional changes between gene expression in THY and AF. A. Bar graphs present the number of genes better expressed in THY or AF in three of the most prominently changed metabolic categories. B. Major metabolic trends in nutrient acquisition. Red symbolizes genes better expressed in AF, green better expressed in THY.

For ease of analysis and description, we assigned the GBS genes to functional categories based on their annotation and presumed involvement in metabolic processes or cell maintenance functions (Tables 1, 2, 3, 4, 5, 6 and Table S1). In general, the transcript levels of most genes in each functional category were better expressed during growth in THY. However, the transcripts of genes belonging to several functional categories, including amino acid, carbohydrate, and nucleotide metabolism were higher in AF at one or more growth points (Fig. 5A).

**Table 1:** Selected genes involved in stress response regulated in response to amniotic fluid.

Locus	Name	ML	LL	S	Putative function
gbs0009			**−7.2**		Heat shock protein 15
gbs0015	ftsH		**−9.0**	**−4.5**	Cell division protein ftsH (EC 3.4.24.-)
gbs0095	grpE		**−3.7**		GrpE protein
gbs0097	dnaJ			**−6.3**	chaperone protein
gbs0104	tig		**−5.3**		Trigger factor, ppiase (EC 5.2.1.8)
gbs0109	radA		**−8.5**	**−2.4**	DNA repair protein RadA
gbs0222	-		**3.2**	**−3.1**	DNA-damage-inducible protein J
gbs0284	-		**−2.3**		Thioredoxin reductase (EC 1.8.1.9)
gbs0289	recU		**−14.7**	**−2.8**	Recombination protein recU
gbs0447	recX		**−6.3**		Regulatory protein recX
gbs0502			**−5.0**		ATP-dependent endopeptidase Lon (EC 3.4.21.53)
gbs0547	recN		**−2.4**		DNA repair protein recN
gbs0786	recR		**−2.1**	**4.3**	Recombination protein recR
gbs0838	nrdH		**−5.6**		Glutaredoxin
gbs1202	-	**−2.4**			General stress protein, Gls24 family
gbs1257			**−2.6**		Endopeptidase, M10 family (EC 3.4.24.-)
gbs1376	clpL	**−3.3**		**5.2**	ATP-dependent endopeptidase clp ATP-binding subunit clpL
gbs1383	clpX		**−3.9**		ATP-dependent endopeptidase clp ATP-binding subunit clpX
gbs1423	trxB		**−2.6**		Thioredoxin reductase (EC 1.8.1.9)
gbs1586			**−3.6**		Peptidyl-prolyl cis-trans isomerase (EC 5.2.1.8)
gbs1674	-		**2.4**	**2.9**	Endopeptidase htpX (EC 3.4.24.-)
gbs1721	-	**−2.3**	**2.2**		Universal stress protein family
gbs1738	-	**−2.1**		**−2.4**	General stress protein, Gls24 family
gbs1764	mutS2		**−2.8**		DNA mismatch repair protein mutS
gbs1778			**−14.2**	**−27.0**	Universal stress protein family
gbs1865	hslO		**−8.0**		33 kDa chaperonin
gbs2029	groEL		**2.2**		60 kDa chaperonin GROEL
gbs2048	cinA			**2.3**	competence/damage-inducible protein CinA
gbs2052	mutL		**−2.4**		DNA mismatch repair protein mutL
gbs2053	csp		**−55.0**	**−6.0**	Cold shock protein
gbs2054	mutS		**−3.1**		DNA mismatch repair protein mutS
gbs2113	-		**−4.8**		Non-proteolytic protein, peptidase family M16
gbs2115	recF		**−4.9**		DNA replication and repair protein recF

Values represent fold change in expression in amniotic fluid compared to expression in THY; ML, mid-logarithmic growth phase; LL, late-logarithmic growth phase; S, stationary growth phase; cut-off two fold change with P value less than 0.05. Positive values represent genes up-regulated in AF, negative values represent down-regulated (better expressed in THY) genes. Full list of changes is published as Table S1.

**Table 2:** Selected regulators differentially expressed in response to AF.

Locus	Name	ML	LL	S	Putative function
gbs0094	hrcA		**−2.2**		Heat-inducible transcription repressor hrcA
gbs0105	rpoE		**−17.7**		DNA-directed RNA polymerase delta chain (EC 2.7.7.6)
gbs0118	-	**−5.8**			Ribose operon repressor
gbs0121			**−2.3**	**−2.6**	Two-component response regulator
gbs0135	mecA		**−7.9**		Negative regulator of genetic competence mecA
gbs0156	rpoB		**−3.9**		DNA-directed RNA polymerase beta chain (EC 2.7.7.6)
gbs0181	-		**−3.9**		Autolysin response regulator
gbs0248	-	**−2.3**	**−4.4**		ECF-type sigma factor negative effector
gbs0249	-	**−3.4**	**−17.4**		RNA polymerase ECF-type sigma factor
gbs0299			**−4.4**		Two-component response regulator
gbs0302	-		**−9.2**		DNA-directed RNA polymerase omega chain (EC 2.7.7.6)
gbs0414	nusA			**−2.6**	transcription elongation factor NusA
gbs0427	perR		**−2.9**		Oxidative stress response regulator BosR
gbs0429		**−2.0**	**−2.9**		Two-component response regulator SaeR
gbs0546	argR1		**−4.0**	**−2.6**	Arginine repressor, argR
gbs0680	ccpA			**−4.4**	catabolite control protein A
gbs0741	vicR		**−2.2**		Two-component response regulator VicR
gbs0756			**−2.6**		Stress-responsive transcriptional regulator PspC
gbs0804			**−3.5**		Catabolite control protein B
gbs1050	-	**4.4**	**4.2**	**13.3**	Carbon starvation protein A
gbs1398	-	**−2.2**	**−6.8**		Two-component response regulator
gbs1496	rpoD		**−2.1**	**−2.8**	RNA polymerase sigma factor rpoD
gbs1530	rofA			**−5.3**	transcriptional regulator
gbs1672	covR		**−2.3**		Response regulator CsrR
gbs1719	codY		**−11.0**		Transcription pleiotropic repressor codY
gbs1736	scrR	**−4.3**			Sucrose operon repressor
gbs1835			**−2.6**	**−3.1**	Transcriptional regulatory protein
gbs1870	ctsR		**−4.7**		Transcriptional regulator ctsR
gbs1882	-		**−2.4**		Catabolite gene activator
gbs1909	dpiA		**−4.6**	**−18.6**	Transcriptional regulatory protein
gbs1934	-	**−2.7**	**−20.4**	**−13.4**	Two-component response regulator yesN
gbs1944	fasA	**2.0**			Response regulator FasA
gbs1948			**−2.6**		Alkaline phosphatase synthesis two-component response regulator phoP
gbs2055	argR2		**−4.0**		Arginine repressor, argR
gbs2081			**−2.3**		Transcriptional regulatory protein
gbs2087		**−3.8**	**−6.9**	**−10.8**	Two-component response regulator
gbs2119	ahrC.2		**−8.3**	**−3.3**	Arginine repressor, argR

Values represent fold change in expression in amniotic fluid in comparison with expression in THY. Positive values represent genes up-regulated in AF, negative values represent down-regulated (better expressed in THY) genes. ML, mid-logarithmic growth phase; LL, late-logarithmic growth phase; S, stationary growth phase; cut-off: fold change above 2 and P value less than 0.05. Full list of changes is published as Table S1.

**Table 3:** Selected genes involved in pathogenesis up- or down-regulated (better expressed in THY) in response to amniotic fluid.

Locus	Name	ML	LL	S	Region	Putative function
gbs0031			**−6.9**			Surface antigen
gbs0393		**−3.5**	**−4.2**	**−3.2**		Hypothetical protein
gbs0451		**3.2**	**−2.6**			C5A peptidase precursor
gbs0470	alp2	**−2.3**		**−2.8**		Cell surface protein
gbs0631				**2.9**		Sortase
gbs0644	cylX		**4.6**		H	Hypothetical protein
gbs0645	cylD	**2.6**	**5.5**		H	Malonyl-CoA-transacylase
gbs0646	cylG	**3.7**	**6.8**		H	3-oxoacyl-reductase
gbs0647	acpC	**3.9**	**4.3**		H	Acyl carrier protein
gbs0648		**2.7**	**4.0**		H	(3R)-hydroxymyristoyl-dehydratase
gbs0649	cylA	**2.8**	**4.2**		H	ATP-binding protein
gbs0650	cylB	**3.6**	**2.5**		H	Permease
gbs0651	cylE	**3.4**	**2.3**		H	Hypothetical protein
gbs0652	cylF	**3.8**	**2.4**		H	Aminomethyltransferase
gbs0653	cylI	**4.0**	**2.3**		H	3-oxoacyl synthase
gbs0654	cylJ	**3.9**	**2.2**		H	UDP glycosyltransferase
gbs0655	cylK	**2.7**	**2.1**	**−2.5**	H	Hypothetical protein
gbs0850	fbp	**−5.1**				Hypothetical protein
gbs1061		**4.1**			I	Hypothetical protein
gbs1062		**3.8**			I	Hypothetical protein
gbs1064		**2.3**			I	Hypothetical protein
gbs1065		**2.6**			I	Hypothetical protein
gbs1066		**3.5**			I	Hypothetical protein
gbs1067		**3.2**			I	Hypothetical protein
gbs1068		**3.2**			I	DNA segregation ATPase
gbs1069		**5.7**			I	Hypothetical protein
gbs1070		**5.1**	**3.9**	**5.7**	I	Hypothetical protein
gbs1071		**4.8**	**3.8**		I	Hypothetical protein
gbs1072		**4.7**	**4.5**		I	Hypothetical protein
gbs1073		**4.1**	**3.3**		I	Phage infection protein
gbs1074		**4.6**	**4.8**		I	Hypothetical protein
gbs1075		**4.9**	**7.5**	**3.9**	I	Hypothetical protein
gbs1076		**3.8**	**6.5**	**7.0**	I	Hypothetical protein
gbs1087		**3.5**	**3.0**			Hypothetical protein
gbs1104		**−2.1**				Antigen
gbs1143	epf	**−3.0**	**−5.4**			Cell surface protein
gbs1144			**−2.3**	**−2.2**	C	Cell surface protein
gbs1234	neuD			**2.7**	C	Sialic acid biosynthesis protein NeuD
gbs1237.1	cpsL		**−2.2**		C	beta-D-Galp alpha-2,3-sialyltransferase
gbs1238	CpsIaJ		**−2.5**		C	beta-D-GlcNAc beta-1,4-galactosyltransferase
gbs1239	hasA		**−5.0**		C	beta-D-Galp beta-1,3-N-acetylglucosaminyltransferase
gbs1240	cpsI	**−2.5**	**−4.9**		C	Secreted polysaccharide polymerase
gbs1241	cpsG		**−3.2**		C	beta-D-Glcp beta-1,4-galactosyltransferase
gbs1242	cpsF		**−3.2**		C	Beta-1,4-galactosyltransferase accessory protein
gbs1248	cpsY		**−2.2**	**−2.5**	C	Transcriptional regulators, LysR family
gbs1307	lmb		**−8.5**			Laminin-binding surface protein
gbs1356				**8.1**		Cell surface protein
gbs1403		**8.1**				5 -nucleotidase
gbs1420		**−2.8**				Choline-binding protein
gbs1474		**−3.6**		**−2.6**		Hypothetical protein
gbs1475		**−4.8**				Sortase
gbs1477				**−10.1**		Cell wall surface anchor family protein
gbs1478		**−2.7**		**−5.1**		Collagen adhesion protein
gbs1492	rgpBc		**−15.1**		B	alpha-L-Rha alpha-1,3-L-rhamnosyltransferase
gbs1493	rgpAc		**−17.3**		B	alpha-D-GlcNAc alpha-1,2-L-rhamnosyltransferase
gbs1494	rmlD		**−9.2**		B	dTDP-4-dehydrorhamnose reductase
gbs1539			**−5.2**	**−3.7**		Hypothetical protein
gbs1926			**−2.6**			Laminin-binding surface protein
gbs1929		**11.7**	**2.7**	**−3.1**		2, 3 -cyclic-nucleotide 2 -phosphodiesterase
gbs2000	cfa		**3.2**			CAMP factor
gbs2008	SpyCEP	**35.8**	**194.8**			Endopeptidase lactocepin

Values represent fold change in expression in amniotic fluid in comparison with expression in THY. ML, mid-logarithmic growth phase; LL, late-logarithmic growth phase; S, stationary growth phase. Cut-off: fold change above 2 and P value below 0.05; Classification of putative virulence genes in GBS (after Glaser, et al, 2002) H – hemolysin, I – pathogenicity island IX, C – capsule, B – group B antigen. Full list of changes is published as Table S1.

**Table 4:** Selected genes involved in peptide and amino acid metabolism and transport differentially expressed in response to amniotic fluid.

GBS	Locus	ML	LL	S	Descriptions
					**Peptidases**
gbs0287	pepC	**3.0**	**4.9**	**5.9**	Aminopeptidase C (EC 3.4.22.40)
gbs1021	pepN		**−3**		Aminopeptidase N (EC 3.4.11.15)
gbs0779	pepF		**2.5**	**4.3**	Oligoendopeptidase F (EC 3.4.24.-)
gbs1459	pepT		**−2.0**	**−3.7**	Tripeptidase T (EC 3.4.11.4)
gbs1751			**−3.8**	**−3.1**	Xaa-Pro dipeptidase (EC 3.4.13.9)
gbs1781	pepXP		**2.7**		Xaa-Pro dipeptidyl-peptidase (EC 3.4.14.11)
					**Oligopeptide transport**
gbs0144	oppA	**2.4**	**2.6**	**3.5**	Oligopeptide-binding system
gbs0145	oppB		**6.1**		Oligopeptide transport system
gbs0146	oppC		**8.0**		Oligopeptide transport system
gbs0147	oppD	**2.2**	**9.0**	**4.1**	Oligopeptide transport system
gbs0148	oppF	**2.1**	**9.7**	**2.6**	Oligopeptide transport system
gbs0966	oppA		**23.2**		Oligopeptide-binding protein oppA
gbs1513			**−2.5**		Di- tripeptide transporter
					**Branched chains amino acids**
gbs1627		**16.3**	**319.3**		
gbs1628		**14.5**	**502.1**		ATP-binding protein livF
gbs1629		**17.7**	**146.5**		ATP-binding protein livG
gbs1630		**16.9**	**227.8**		permease protein livM
gbs1631		**19.7**	**94.9**		permease protein livH
gbs1632		**8.6**	**92.9**		binding protein
gbs1683	braB	**5.77**			Branched-chain amino acid transport
gbs2004			**8.2**		
gbs2005		**2.1**	**4.2**		
gbs2006			**5.4**		Branched-chain amino acid transport
gbs2007		**2.7**	**5.2**	**6.8**	Branched-chain amino acid transport
gbs1610		**3.4**	**10.0**		carrier protein
					**Arginine**
gbs1077	carB	**6.0**	**7.3**		Carbamoyl-phosphate synthase large chain
gbs1078	carA	**6.3**	**7.5**		Carbamoyl-phosphate synthase small chain
gbs2122	arcA		**2.7**	**2.1**	Arginine deiminase (EC 3.5.3.6)
gbs2123				**4.1**	Acetyltransferase (EC 2.3.1.-)
gbs2124	arcB			**5.3**	Ornithine carbamoyltransferase (EC 2.1.3.3)
gbs2125	-			**5.5**	Arginine ornithine antiporter
gbs2126	arcC		**−2.1**	**3.6**	Carbamate kinase (EC 2.7.2.2)
gbs2083	-	**−7.5**	**−140.4**	**−31.7**	Arginine ornithine antiporter
gbs2084	arcC	**−3.6**	**−75.0**	**−43.6**	Carbamate kinase
gbs2085	arcB	**−2.3**	**−28.8**	**−19.7**	Ornithine carbamoyltransferase

Values represent fold change in expression in amniotic fluid compared to expression in THY; ML, mid-logarithmic growth phase; LL, late-logarithmic growth phase; S, stationary growth phase; cut-off two fold change with P value less than 0.05. Positive values represent genes up-regulated in AF, negative values represent down-regulated (better expressed in THY) genes. Full list of changes is published as Table S1

**Table 5:** Selected genes involved in nucleotide metabolism regulated in response to amniotic fluid.

GBS	Locus	ML	LL	S	Descriptions
gbs0014			**−8.2**		Hypoxanthine-guanine phosphoribosyltransferase
gbs0017	prsA.2		**−6.1**	**−2.8**	Ribose-phosphate pyrophosphokinase
gbs0023	-	**32.8**	**28.0**		Phosphoribosylaminoimidazole-succinocarboxamide synthase
gbs0024	-	**38.9**	**56.1**		Phosphoribosylformylglycinamidine synthase
gbs0025	purF	**25.1**	**30.7**		Amidophosphoribosyltransferase
gbs0026	purM	**25.6**	**31.8**	**5.4**	Phosphoribosylformylglycinamidine cyclo-ligase
gbs0027	purN	**32.0**	**41.8**		Phosphoribosylglycinamide formyltransferase
gbs0028	-	**21.0**	**26.8**	**9.1**	Zwittermicin A resistance protein zmaR
gbs0029	purH	**27.6**	**29.3**	**12.4**	Phosphoribosylaminoimidazolecarboxamide formyltransferase
gbs0042	purD	**36.4**	**30.0**		Phosphoribosylamine-glycine ligase
gbs0043	purE	**49.0**	**47.5**		Phosphoribosylaminoimidazole carboxylase carboxyltransferase subunit
gbs0044	purK	**34.9**	**27.2**		Phosphoribosylaminoimidazole carboxylase NCAIR mutase subunit
gbs0047	purB	**3.0**	**3.6**		Adenylosuccinate lyase
gbs0106	-		**−12.0**		CTP synthase (EC 6.3.4.2)
gbs0553	pyrD	**6.5**	**3.5**	**7.1**	Dihydroorotate dehydrogenase (EC 1.3.3.1)
gbs0558				**−6.5**	dGTP triphosphohydrolase
gbs0574		**−7.3**			Inosine-uridine preferring nucleoside hydrolase (EC 3.2.2.1)
gbs0583		**4.4**			Adenosine deaminase
gbs0836	nrdF.2		**−3.2**		Ribonucleoside-diphosphate reductase beta chain
gbs0837	nrdE.2		**−3.3**		Ribonucleoside-diphosphate reductase alpha chain
gbs0844	udk		**−29.6**		Uridine kinase
gbs1079	pyrB	**7.1**	**4.5**		Aspartate carbamoyltransferase
gbs1080	pyrC	**3.7**	**3.6**		Dihydroorotase
gbs1081	pyrE	**4.7**	**2.7**		Orotate phosphoribosyltransferase
gbs1082	pyrF	**5.6**	**2.3**	**3.9**	Orotidine 5 -phosphate decarboxylase
gbs1089	fhs.1	**3.1**	**4.1**		Formate-tetrahydrofolate ligase
gbs1110	-		**−5.0**		Thymidine kinase
gbs1116			**2.9**	**−2.6**	Xanthine permease
gbs1117	xpt		**3.1**		Xanthine phosphoribosyltransferase
gbs1154	guaC	**2.4**	**8.8**		GMP reductase
gbs1162	-		**−6.0**	**4.1**	GTP pyrophosphokinase homolog
gbs1231	ung		**−2.7**		Uracil-DNA glycosylase
gbs1867	-		**−13.0**		Deoxyadenosine kinase
gbs1929	-	**11.7**	**2.7**	**−3.1**	2, 3 -cyclic-nucleotide 2 -phosphodiesterase

Values represent fold change in expression in amniotic fluid compared to expression in THY; ML, mid-logarithmic growth phase; LL, late-logarithmic growth phase; S, stationary growth phase; cut-off two fold change with P value less than 0.05. Positive values represent genes up-regulated in AF, negative values represent down-regulated (better expressed in THY) genes. Full list of changes is published as Table S1.

**Table 6:** Selected genes involved in carbohydrate metabolism and transport differentially expressed in response to amniotic fluid.

Locus	Name	ML	LL	S	Putative function
gbs0113		**−2.5**		**9.5**	D-ribose-binding protein
gbs0114		**−2.3**	**2.1**	**9.7**	Ribose transport system permease protein rbsC
gbs0115			**2.7**	**5.7**	Ribose transport ATP-binding protein rbsA
gbs0116			**2.4**	**6.2**	D-ribose mutarotase
gbs0117			**2.5**	**3.0**	Ribokinase
gbs0316	-	**−3.4**	**10.8**		PTS system, cellobiose-specific IIA component
gbs0317	-	**−3.1**	**5.5**	**18.2**	PTS system, cellobiose-specific IIB component
gbs0318			**4.3**		PTS system, cellobiose-specific IIC component
gbs0346	manN	**2.2**	**2.7**	**4.2**	PTS system, mannose-specific IID component
gbs0347	manM			**3.0**	PTS system, mannose-specific IIC component
gbs0348	manL	**2.1**	**2.0**	**2.2**	PTS system, mannose-specific IIAB component
gbs0872	glgC	**2.1**			Glucose-1-phosphate adenylyltransferase catalytic subunit
gbs0873	-	**2.5**			Glucose-1-phosphate adenylyltransferase regulatory subunit
gbs1329	lacG		**−17.1**	**2.3**	6-phospho-beta-galactosidase
gbs1330	lacE		**−8.1**		PTS system, lactose-specific IIBC component
gbs1331	lacF		**−11.7**		PTS system, lactose-specific IIA component
gbs1333	lacD.2		**−12.7**		Tagatose-bisphosphate aldolase
gbs1334	lacC.2	**−2.8**	**−18.7**		Tagatose-6-phosphate kinase
gbs1335	lacB.1	**−2.2**	**−10.2**	**3.9**	Galactose-6-phosphate isomerase lacB subunit
gbs1336	lacA.2	**−2.1**	**−11.2**	**3.1**	Galactose-6-phosphate isomerase lacA subunit
gbs1507	glgP	**−2.4**	**5.1**		Maltodextrin phosphorylase
gbs1508	malM	**−2.9**	**5.9**		4-alpha-glucanotransferase
gbs1510	malE		**3.8**		Maltose maltodextrin-binding protein
gbs1511	malF		**4.6**		Maltodextrin transport system permease protein malC
gbs1512	malG		**3.5**		Maltose transport system permease protein malG
gbs1692		**−2.7**			Dihydroxyacetone kinase
gbs1694		**−2.5**	**5.1**	**5.9**	Dihydroxyacetone kinase
gbs1695			**3.9**	**8.4**	Dihydroxyacetone kinase
gbs1696			**2.6**	**14.5**	Dihydroxyacetone kinase phosphotransfer protein
gbs1697		**−2.4**	**2.5**	**11.8**	Glycerol uptake facilitator protein
gbs1714				**3.9**	Pyruvate,phosphate dikinase
gbs1732	pmi		**−3.2**		Mannose-6-phosphate isomerase
gbs1733	scrK	**−6.9**			Fructokinase
gbs1734	scrA	**−22.3**	**2.6**		PTS system, sucrose-specific IIABC component
gbs1735	scrB	**−5.2**			Sucrose-6-phosphate hydrolase
gbs1777	glpF.2		**2.2**	**3.8**	Glycerol uptake facilitator protein
gbs1797			**−2.3**		Galactose-1-phosphate uridylyltransferase
gbs1811	plr	**2.1**	**2.5**		Glyceraldehyde 3-phosphate dehydrogenase
gbs1850	-	**−2.5**	**−9.9**		Transaldolase
gbs1893	-	**−11.6**	**−6.5**		2-dehydro-3-deoxygluconokinase
gbs1911	dexB		**2.9**		Glucan 1,6-alpha-glucosidase
gbs1912				**2.9**	Multiple sugar transport ATP-binding protein msmK
gbs1914	-	**−7.3**	**−169.1**		Aldose 1-epimerase family protein
gbs1915	-	**−3.3**	**−61.2**	**−2.5**	Tagatose-bisphosphate aldolase
gbs1916	-	**−3.1**	**−92.2**		Tagatose-6-phosphate kinase
gbs1917	-	**−3.1**	**−67.0**		Galactose-6-phosphate isomerase lacB subunit
gbs1918	lacA.1	**−4.2**	**−296.9**		Galactose-6-phosphate isomerase lacA subunit
gbs1919			**−14.8**		Sialidase A precursor
gbs1920			**−10.5**		PTS system, galactose-specific IIC component
gbs1921	-	**−2.3**	**−14.4**		PTS system, galactose-specific IIB component
gbs1922	-	**−3.6**	**−19.2**		PTS system, galactose-specific IIA component
gbs1923	lacR.1	**−2.7**	**−3.4**		Lactose phosphotransferase system repressor
gbs1936	ptsD		**−36.4**	**−10.1**	PTS system, mannose fructose family IID component
gbs1937	ptsC		**−45.8**	**−31.4**	PTS system, mannose fructose family IIC component
gbs1938	ptsB		**−37.6**	**−8.8**	PTS system, mannose fructose family IIB component
gbs1939	-	**−11.1**	**−34.6**	**−42.6**	PTS system, mannose fructose family IIA component
gbs1946			**5.8**	**4.6**	PTS system, glucose-specific IIABC component
gbs2116	-		**−3.2**	**2.3**	Glucose uptake family protein

Values represent fold change in expression in amniotic fluid compared to expression in THY; ML, mid-logarithmic growth phase; LL, late-logarithmic growth phase; S, stationary growth phase; cut-off two fold change with P value less than 0.05. Positive values represent genes up-regulated in AF, negative values represent down-regulated (better expressed in THY) genes. Full list of changes is published as Table S1

### Stress response to AF

We hypothesized that growth in amniotic fluid will trigger expression of genes involved in adaptation and the stress response. Although we observed changes in the transcript levels of multiple genes involved in adaptation, protein secretion and trafficking, and DNA repair, surprisingly, the transcripts were down regulated in response to growth in AF (Table 1). The most striking examples are genes encoding putative cold shock protein (*gbs2053*, 55× down regulated in AF) or universal stress protein family (*gbs1778*, 27× down regulated in AF). However, we also observed moderate up-regulation of *groEL* and *clpL* transcripts. Thus, it appears that GBS does not exhibit a classic stress response when grown in AF, but rather readily adapts to this environment. We also did not observe massive down regulation of protein synthesis as an effect of stress, and interestingly, production of some ribosomal protein transcripts in LL and S phase was higher in AF than in THY (Table S1).

### Regulatory events during growth in AF

The lack of alternative sigma factors in GBS [10] means that a successful regulatory response to environmental changes relies mainly on differential transcription of genes encoding two component systems (TCS) and stand-alone regulators. The GBS genome has genes encoding multiple TCS systems that might be involved in adaptation to various environments. As expected, we observed differential expression of multiple TCS and putative regulators of unknown function (Table 2). One of the regulators with the greatest degree of transcriptional change encodes carbon starvation protein A (Table 2). This change, together with observed alteration in transcripts of the genes encoding carbon catabolite proteins A and B might contribute to the large number of differential transcripts observed in genes encoding metabolic proteins (see below). In addition, we observed lowered expression of *codY*, a regulator involved predominantly in amino acid metabolism and activated by branched chain amino acids [22]. In *Streptococcus pneumoniae* inactivation of *codY* gene is linked to decreased expression of *pcpA* adhesin and lower adhesion to human cells in vitro, suggesting possible mechanism linking metabolic state of the bacterium and pathogenic properties [23]. Recent analysis of *Streptococcus pyogenes* adaptation to blood found that a *codY* mutant strain strongly up-regulated expression of genes encoding branched chain amino acids transporters [24], an observation consistent with differential expression of branched chain amino acid transporter genes we found in our experiment. Among differentially regulated TCSs we noted changes in expression of Gbs1671/1672 TCS, a homolog of the GAS CovR/S that is a major negative regulator of virulence genes in GBS and GAS [25], [26]. We also observed differential expression of Gbs1397/1398, a TCS with ∼78% similarity to the GAS SptRS system (Spy874/875) required for survival in saliva and an important regulator of virulence and carbohydrate utilization GAS [9], [12]. These GBS genes may regulate genes affecting carbohydrate metabolism.

### Expression of virulence factors

Compared to other pathogenic streptococci such as GAS, virulence factors of GBS are much less studied and therefore not well understood. However, we observed differential expression of multiple putative cell wall anchored proteins (Table 3) and proven virulence factors. For example, the *cyl* operon, encoding a hemolysin, required for survival in blood and under oxidative stress [27], [28] was up-regulated during growth in human AF. One of the more striking observations was very high up-regulation of the homolog of GAS SpyCEP (*gbs2008*). This extracellular protease cleaves and inactivates human interleukin 8 and contributes to virulence in GBS [29] and GAS [30], [31]. In GAS, SpyCEP is greatly up-regulated in strains causing invasive infections compared to those recovered from patients with superficial infections such as pharyngitis [31]. We also detected an increased level of transcripts encoded by genes located in putative pathogenicity island IX (gbs1061-gbs1076), function of this element is unknown. Interestingly, transcripts of genes encoding multiple proteins implicated in adhesion to host molecules such as fibronectin, collagen, and laminin were significantly down-regulated during growth in AF. The C5a peptidase gene transcript also was significantly lower in GBS grown in AF, consistent with its involvement in fibronectin binding [32]. Down-regulation of adhesins seems to be consistent with increased virulence. For example, molecular epidemiological data suggest a negative correlation between binding of fibronectin and severity of GAS infection [33]. Over-expression of fibronectin binding protein decreases the virulence of GAS lacking fibronectin binding protein gene resulted in reduced virulence and lack of fibronectin binding protein the surface promotes bacterial dissemination [34].

### Metabolism: Nutrient acquisition and energy production

GBS requires multiple exogenous compounds for growth, especially amino acids (AA). AF is composed mostly of water, urea, small amounts of amino acids, keratin from shed host epithelial cells, and proteins [35], [36], [37]. Thus, AF is relatively poor in nutrients, which means that bacteria with complex nutritional requirements will not grow or grow poorly. Unexpectedly, strain NEM316 grew very rapidly and to high cell density in AF (Fig. 1). We discovered that genes encoding systems that facilitate transport of amino acids and peptides were prominently up-regulated when GBS was grown in AF (Figure 5AB). In particular, multiple transport systems for branched-chain amino acids (isoleucine, leucine, valine) were very highly up-regulated, on the order of up to 500× more highly expressed during growth in THY (Table 4). Recently Samen and co-workers [38] showed that growth of GBS in AF depends on intact isoleucine and oligopeptide transport systems. Because GBS is auxotrophic towards multiple amino acids, presumably the up-regulation of amino acid transport systems is a direct result of an effort to scavenge these molecules. Oligopeptide transport systems in group A and B streptococci has been also shown to be involved in pathogenic properties as adhesion [38], [39]. Recently, increasing number of reports links ability to utilize nutrients and metabolic state of the bacterium with its pathogenic properties [13], [40]. Therefore, similar to carbohydrates, amino acid and oligopeptide transport and utilization processes might play a role in pathogenicity of GBS.

We also observed significant differential expression of genes in the arginine deiminase pathway. Arginine fermentation can be used for energy production by GAS, and likely GBS [41]. The genome of GBS strain NEM316 has two putative sets of genes involved in this metabolic pathway. However, it has not been confirmed experimentally if both of them are indeed involved in arginine deiminase pathway. Locus 1 (with high homology to GAS) is up regulated in response to AF, locus 2 with lower homology to GAS genes is down regulated in response to AF. Arginine deiminase seems to have a profound effect on streptococcal biology and virulence. We recently described regulation of arginine deiminase by growth phase [5] in GBS, and a similar phenomenon was also described recently for *Streptococcus gordonii* [42]. Arginine deiminase also influences expression of fimbriae in *Porphyromonas gingivalis* [43]. In GAS, arginine utilization is under control of major regulators *rgg* [44] and *ccpA* [13] and arginine deiminase is a potent inhibitor of human T-cell proliferation [45]. Moreover, despite the fact that it lacks export signal sequence, arginine deiminase is found on the GAS cell surface [46] and is a protective antigen in mice [A. Henningham, M.R Batzloff, J.C. Cole, C.M. Gillen, J. Hartas, K. S. Sriprakash, M. J. Walker, poster nr. P54, 2008 Lancefield International Symposium on Streptococci & Streptococcal Diseases, Porto Heli, Greece].

Additionally, arginine metabolism is linked to nucleotide metabolism by carbamoyl phosphate. The *carAB* genes encoding subunits of carbamoyl phosphate synthase are also differentially regulated in GBS grown in AF. We observed significant up regulation of gene transcripts for almost all enzymes involved in *de novo* purine and pyrimidine synthesis and down regulation of salvage pathways (Table 5). This was especially prominent in the early stages of growth, suggesting that AF lacks sufficient free nucleotides for rapid growth.

### Carbohydrate metabolism

Among genes involved in carbohydrate utilization in GBS, large portion is constituted by multiple transport systems (mostly PTS) that allow uptake of various carbohydrate sources. We detected differential expression of many genes involved in carbohydrate transport and metabolism (Table 6) (Fig. 5B). Interestingly, GBS rather down-regulated genes responsible for the transport of simple carbohydrates what suggests their low concentration in AF.

### Summary

We have discovered that GBS significantly remodels its transcriptome in response to exposure to human amniotic fluid. A large number of the affected genes are of unknown function, which means that much remains to be learned about the full influence of amniotic fluid on GBS. The majority of the observed changes in transcripts affects genes involved in basic bacterial metabolism and is connected to AF composition and nutritional requirements of the bacterium. The observation that many genes encoding adhesions are down-regulated, and genes encoding known virulence factors such as a hemolysin and a potent IL-8 proteinase are up-regulated likely have consequences for the outcome of host-pathogen interactions.

## Supporting Information

Table S1 Changes in transcription of GBS genes upon contact with amniotic fluid. All changes detected in transcription of GBS in response to amniotic fluid Values represent fold change in expression in amniotic fluid compared to expression in THY; ML, mid-logarithmic growth phase; LL, late-logarithmic growth phase; S, stationary growth phase; cut-off two fold change with P value less than 0.05. Positive values represent genes up-regulated in AF, negative values represent down-regulated (better expressed in THY) genes.
(0.26 MB XLS)
